# Relationship between asymmetric nostril use and human emotional odours in cats

**DOI:** 10.1038/s41598-023-38167-w

**Published:** 2023-07-06

**Authors:** Serenella d’Ingeo, Marcello Siniscalchi, Valeria Straziota, Gianluca Ventriglia, Raffaella Sasso, Angelo Quaranta

**Affiliations:** Animal Physiology and Behaviour Unit, Department of Veterinary Medicine, University of Bari Aldo Moro, 70121 Bari, Italy

**Keywords:** Cognitive neuroscience, Emotion

## Abstract

Cat social behaviour and cognition has received a growing interest during the last decades. Recent studies reported that cats efficiently engage in interspecific communication with humans and suggest that cats are sensitive to human emotional visual and auditory cues. To date, there is no evidence on the social and informative role of human emotional odours, which may affect human-cat communication. In this study, we presented cats with human odours collected in different emotional contexts (fear, happiness, physical stress and neutral) and evaluated the animals’ behavioural responses. We found that “fear” odours elicited higher stress levels than “physical stress” and “neutral”, suggesting that cats perceived the valence of the information conveyed by “fear” olfactory signals and regulate their behaviour accordingly. Moreover, the prevalent use of the right nostril (right hemisphere activation) with the increase of stress levels, particularly in response to “fear” odours, provides first evidence of lateralized emotional functions of olfactory pathways in cats.

## Introduction

Despite domestic cats having lived for thousands of years alongside humans^1^, they still retain the morphological, genetic and behavioural characteristics of the wildcat^2^. However, domestication shaped the social behaviour of the cat’s solitary ancestry, which developed the ability to socialize and cooperate with other conspecifics and later with humans for adapting to anthropogenic niche, where the concentration of resources (i.e. food and denning sites) allowed the cohabitation of several individuals^3^. Cat social cognition has received a growing interest in the last decades. The features of human-cat relationship and communication have been investigated highlighting the influence of both ontogenetic and evolutionary factors (e.g., biological predisposition and temperament) on cat social abilities^4–6^. Cats efficiently engage in interspecific communication as they display specific signals directed towards humans (facial expressions^7^ and vocalizations^8,9^) and are capable of recognizing the information content of human cues^10–12^. Several studies demonstrate that cats are able to follow human gestures to locate hidden food (pointing^10^ and gazing^11^) and are sensitive to human ostensive cues and attentional states^11–14^. The human attentional availability significantly affects the expression of human-directed signals in cats, which spend a longer time in proximity with attentive than inattentive humans^13^ and direct more intentional behaviours (i.e. gaze alternation) towards attentive humans in order to access resources out of their reach^14^. Moreover, human actions could significantly bias cats’ behaviour in a two-choice task toward the object the humans interacted with, even though the choice is disadvantageous to the animals (causing the loss of food)^15^. Hence, social stimuli appear to be prioritized when making decisions and are preferred over food and toys by cats^16^.

Beside human visual signals, cats are shown to use human vocal cues for individual recognition^17^ and spatial location^18^ and to acquire social information based on the phonemic features of human utterance^5,19^. It has been found that pet cats differentiate their own names from other words^19^ and distinguish the speech directed to them (i.e. Cat-directed Speech) from speech directed to adult humans (i.e. Adult-directed Speech^5^), particularly when given by their owner. The ability of using and reacting to human communicative signals is fundamental for interspecific interactions and may have substantially contributed to the widespread of domestic cat worldwide, making it one of the most popular pets^20^. The affiliation with humans, however, raises some concerns regarding the influence of human behaviour on cat behaviour and welfare. A recent study investigating this issue found that the owners’ personality traits significantly affect cats’ behaviour. Specifically, high level of owners’ Neuroticism was associated with more aggressive and fearful cat behavioural styles; contrarily, high levels of owner Conscientiousness, Agreeableness, and Openness were associated with less aggressive and aloof cat behavioural styles; whereas high Conscientiousness was related to more gregarious and less fear-related behaviours^21^. Interestingly, owner rating higher Neuroticism reported more behavioural problems of their cats, suggesting that human interaction styles could substantially impact cat wellbeing. Given that cats form stable social bonds with humans that differ among the individuals^6^, the relationship between owner personality and human-cat bond style warrants further investigation.

Recent evidence suggests that cats are sensitive to human emotional cues, which strongly modulate the interactions between individuals in social species^22^. Galvan and Vonk^23^ have found that cats respond more positively to their owners when they express facial and postural signals of happiness than anger. In particular, cats were more likely to engage in positive behaviours (e.g. ears forward or normal, relaxed body posture) and spent a longer time in contact with their owners when they appeared happy. However, given the subtle changes of the animals’ behaviour registered in the study, the authors concluded that cats are only modestly affected by human emotional cues. Similarly, Merola and colleagues^24^ found that the owners’ emotional expression slightly affects cats’ reaction toward an unfamiliar and potentially frightening object. Although the likelihood and frequency of gazing at human face (known as “social referencing behaviour”) was higher when the owners expressed a negative reaction to the objects, only subtle differences in cats’ behaviour were observed between the positive and negative emotional conditions (i.e. more static behaviour registered in the positive context). Nevertheless, a growing body of literature provides considerable evidence of cats’ ability to perceive and functionally respond to human emotional states. A recent study shows that cats not only recognize human emotions of anger and happiness by correctly matching vocalization to facial expressions but they functionally respond to the valence of the emotion perceived^25^, showing higher stress levels when the anger face/vocalization were presented compared to happiness ones. Human emotional states appear also to influence human-directed social behaviour of cats: they engaged in more head- and flank-rubbing behaviour toward depressive owners and approached more extroverted or agitated owners than those feeling numb^26,27^. Furthermore, cats prefer to approach humans giving a slow blink stimulus, which is produced in calm and positive context^28^, compared to those displaying a neutral facial expression^29^. This preference has been related to the cats’ perception of the positive content of the human relaxation signal, which in turn elicited a positive emotional state in cats, as they responded by producing eye narrowing movements their own^29^. Overall, these findings suggest that cats engage in emotional communication with humans.

Olfaction plays an important role in the social lives of domestic cats. It is used to maintain space between individuals (to avoid territorial overlapping) and sustains the cohesion of colony members, providing social information about conspecifics^3,20^. Among the affiliative behaviours of cats, mutual allorubbing and allogrooming, which involve the exchange of scents between the individuals, are commonly displayed. Interestingly, cats appear to direct allorubbing also to humans in contexts similar to conspecific interactions. It is therefore hypothesised that human-directed allorubbing could retain the meaning of cat-to-cat communication^3^. However, evidence of human-cat olfactory communication is still scarce. Recent studies evaluating the presence of lateralized behaviours for sniffing emotional odours reported functional asymmetries in emotional processing in both dogs and horses^30–32^. Specifically, the preferential use of a nostril, which indirectly reflects the prevalent activation of the ipsilateral brain hemisphere, has been observed. Considering that brain hemispheres have different specializations for emotional functioning, the analysis of the nostril preferential use provides indirect information on subjects’ arousal levels and the valence of the emotion experienced by each individual^33^. Specifically, the left hemisphere regulates the expression of positive emotions, pro-social and approach behaviours, whereas the right hemisphere is mainly involved in the processing of arousing stimuli and the expression of intense emotions (i.e. fear and anger)^33,34^. To date, there is no evidence on asymmetric nostril use during sniffing behaviour in cats. However, previous studies report the presence of functional lateralization for emotional processing in this species, particularly for acoustic emotional stimuli^35^, suggesting that the expression of lateralized sniffing behaviour could be likely.

Given the crucial role of emotions in human-cat interactions and communication, we investigated cat behavioural responses (including the asymmetries in nostril use) to human odours collected in different emotional contexts.

## Material and methods

### Subjects

Twenty-two cats participated to the study. They were 10 males (9 neutered) and 12 females (11 neutered), whose age ranged between 7 months and 11 years (4.04 ± 3.00; mean ± S.D.). The sample size was determined according to a recent study showing significant differences in cat behavioral responses to human emotional signals (N = 10)^25^. All the tested subjects underwent a clinical evaluation at the Department of Veterinary Medicine to certify the absence of any organic and behavioural disorders (e.g., fear toward unfamiliar humans). Cats were all living indoors and eight of them could access to outdoor areas (i.e., in the countryside) for no more than 5 h per day (typically when owners were not at home). Since the novelty of the testing environment has been shown to affect cats’ behaviour^12^, the experiments were carried out in the cats’ living environment.

### Stimuli

Three healthy men, between the age of 24 and 28 (33.00 ± 7.81; mean ± S.D.), voluntarily participated to the study as donors. Human sweat samples were collected in different emotional conditions, i.e., happiness, fear, physical stress and neutral, following the procedure described in Siniscalchi and colleagues^30^. Briefly, donors had to conform to specific dietary rules and avoid scent products for their personal and clothes hygiene. The sample collection occurred over four consecutive days at the same time (9 a.m.). “Happiness” and “fear” samples were obtained in two sessions by presenting donors with 15-min videos that elicited the related emotions. Donors’ emotional reactions were evaluated through a five-point Visual Analogue Scale (VAS, scores from 1 to 5; see Supplementary Information Fig. S1) that each donor had to fill in at the end of each video, indicating the intensity of happiness and fear felt. The “physical stress” samples were taken after a 15-min run whereas the “neutral” after the morning shower. The emotional odours were collected by placing 3 sterile cotton swabs under each donor’s armpit. Thus, 24 odour samples were obtained for each emotional condition. The samples were immediately stored at − 20 °C (within 1 min from the collection) and defrosted 30 min before the test. During the test, swabs were kept refrigerated to prevent the quality loss of the odours.

### Testing apparatus and procedure

The experiment was carried out in an isolated room of each subject’s home. Before the beginning of the test, cats were allowed to explore and freely interact with the experimenter to become familiar with the experimental set-up. The familiarization phase lasted until cats showed no stress behaviours and not exceeding 20 min.

**Figure 1:**
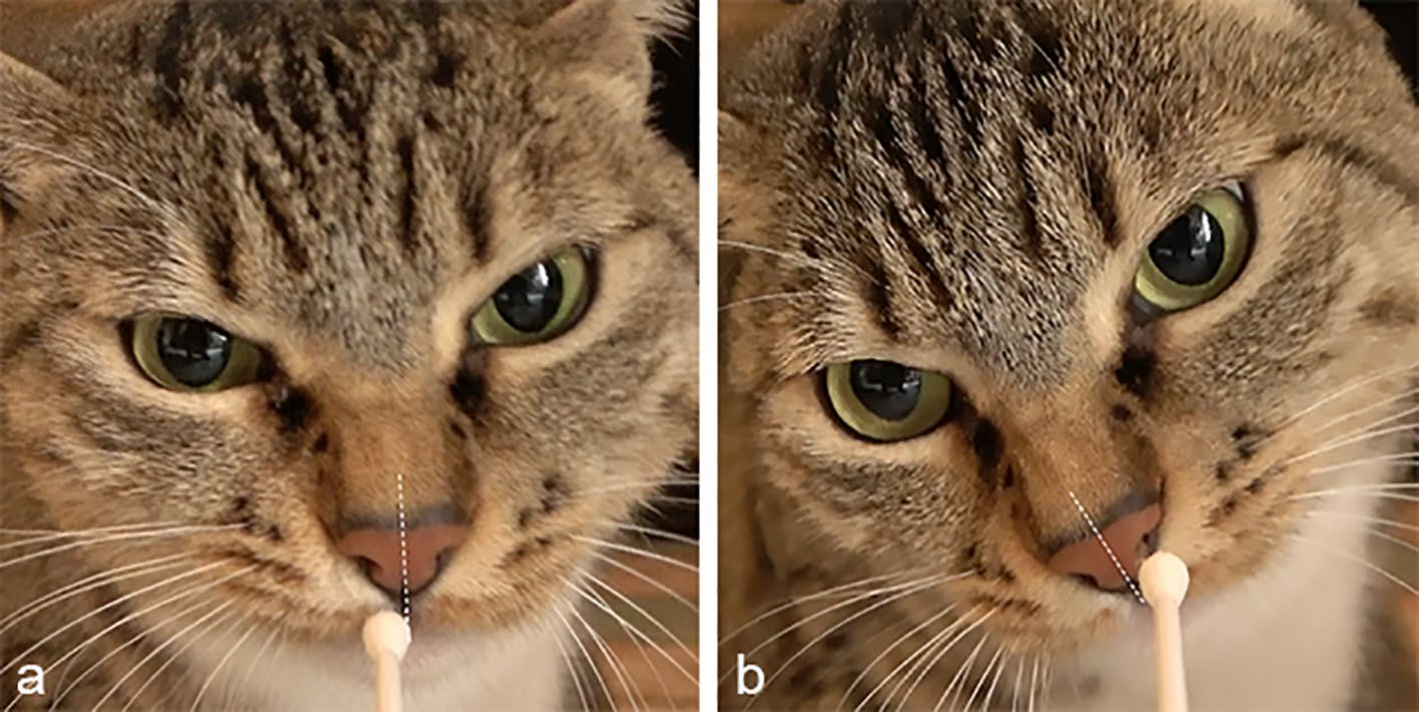
Stimuli presentation to cats. Example of the human emotional odour presentation and the cats’ nostril use: the right (**a**) and the left nostril preferential use (**b**) are shown (dotted line passing through the philtrum).

Human emotional odours were presented only once to each cat in a random order between subjects (but balanced with regard to the emotions). Each cotton swab impregnated with the emotional odour was fixed under a video camera held by the experimenter. The owner and the experimenter sat facing each other and aligned at a distance of 2 m. Cats were called by their owner and gently positioned centrally and in front of the experimenter. Once the animals reached the initial position, they were let free to move and spontaneously approach the swabs (Fig. 1). Each stimulus presentation lasted 45 s but, if no sniffing behaviours occurred within 1 min, the swab was removed and the next stimulus was presented. The inter-stimulus interval was 40 s. During the test, owners were asked not to interact with their cats, particularly through eye contact and vocal cues that could affect the animals’ behaviour. The test was video recorded by two high-resolution cameras (Sony 4K FDR-AX43®), one held by the experimenter and the other placed on a tripod located behind the experimenter at a distance of 1.5 m.

### Data analysis

The video recordings of the test were analysed frame by frame by two trained observers, who were blind to the stimuli presented to the animals. The behavioural analysis was performed using The Observer XT (Noldus®). The frequency of behaviour related to relaxed emotional state, moderate and severe stress was coded (see Supplementary Information Table S1)^36^. Moreover, the nostril used preferentially to sniff human emotional odours was evaluated^30^. In particular, the total time spent sniffing with the right/left nostril was computed when the swab was entirely placed on the right/left of the philtrum (Fig. 1). The asymmetries in nostril use were calculated using the index: LI = (L − R/L + R) where L and R indicate the total time spent sniffing with the left and right nostril, respectively. Therefore, a score of 1.0 indicates the exclusive use of the left nostril, a score of − 1.0 the exclusive use of the right nostril whereas a score of 0 indicates the equal use of both right and left nostril. Finally, the total time spent sniffing the emotional odours was computed. We considered the time spent sniffing with the right/left nostrils and the time spent sniffing the swab when not entirely placed at the right/left of the philtrum (with both nostrils) (see Supplementary Information Fig. S2).

The inter-rater reliability was assessed by means of independent parallel coding of cats’ behaviour during the test and was calculated as the percentage agreement. It was always higher than 95% for each tested variable.

### Statistical analysis

GLMM analysis was performed in order to assess the influence of the emotions (i.e. fear, physical stress, happiness and neutral), stimulus order (and their interactions) on the behavioural categories (i.e. severe and moderate stress, relaxed) and Laterality Index (LI), with subjects as a random variable. Sex and age variables were removed from the analysis as they lowered the predictability of the final model. Since the values of the tested variable were distributed along a positive scale that was skewed toward larger positive values, the inverse Gaussian distribution and log-link function were used. Bayesian information criterion (BIC) was employed for selecting and comparing models based on the − 2 log likelihood. To detect differences between different groups Fisher’s Least Significant Difference (LSD) pairwise comparisons were performed.

Data distribution was tested using Shapiro–Wilk test. According to data distribution, Spearman and Pearson correlations were used to measure the association between the laterality index and the behavioural categories as cumulative data (i.e., severe, moderate stress and relaxed for all the emotional odours presented: fear, physical stress, happiness and neutral) and for each emotional odours; the total time spent sniffing at each emotional odours and the behavioural categories; the cats’ age and both the laterality index and behavioural categories. Differences in the total time spent sniffing the swabs between the emotional odours were tested using a Friedman test. Moreover, pairwise comparisons between the severe and moderate stress for each emotion presented were performed through Wilcoxon signed-rank test. Asymmetries at a group-level in the nostril preferential use were assessed via One-sample Wilcoxon signed-ranks test, to report significant deviation from zero.

Statistical analyses were performed using SPSS® software version 22 (IBM, Armonk, USA, New York).

### Ethics statement

The experiment was conducted according to the protocols approved by the Italian Minister for Scientific Research in accordance with EC regulations and were approved by the Department of Veterinary Medicine (University of Bari) Ethics Committee EC (Approval Number: 19/2020). Written informed consent was obtained from the owners before the beginning of the test. Moreover, written informed consent was obtained from the three human donors. In addition, the study is reported in accordance with ARRIVE guidelines (https://arriveguidelines.org).

## Results

VAS scores of donors collected after watching the emotion-eliciting films were 4.33 ± 0.58 (happiness) and 3.33 ± 0.58 (fear) (mean ± s.d.).

**Figure 2:**
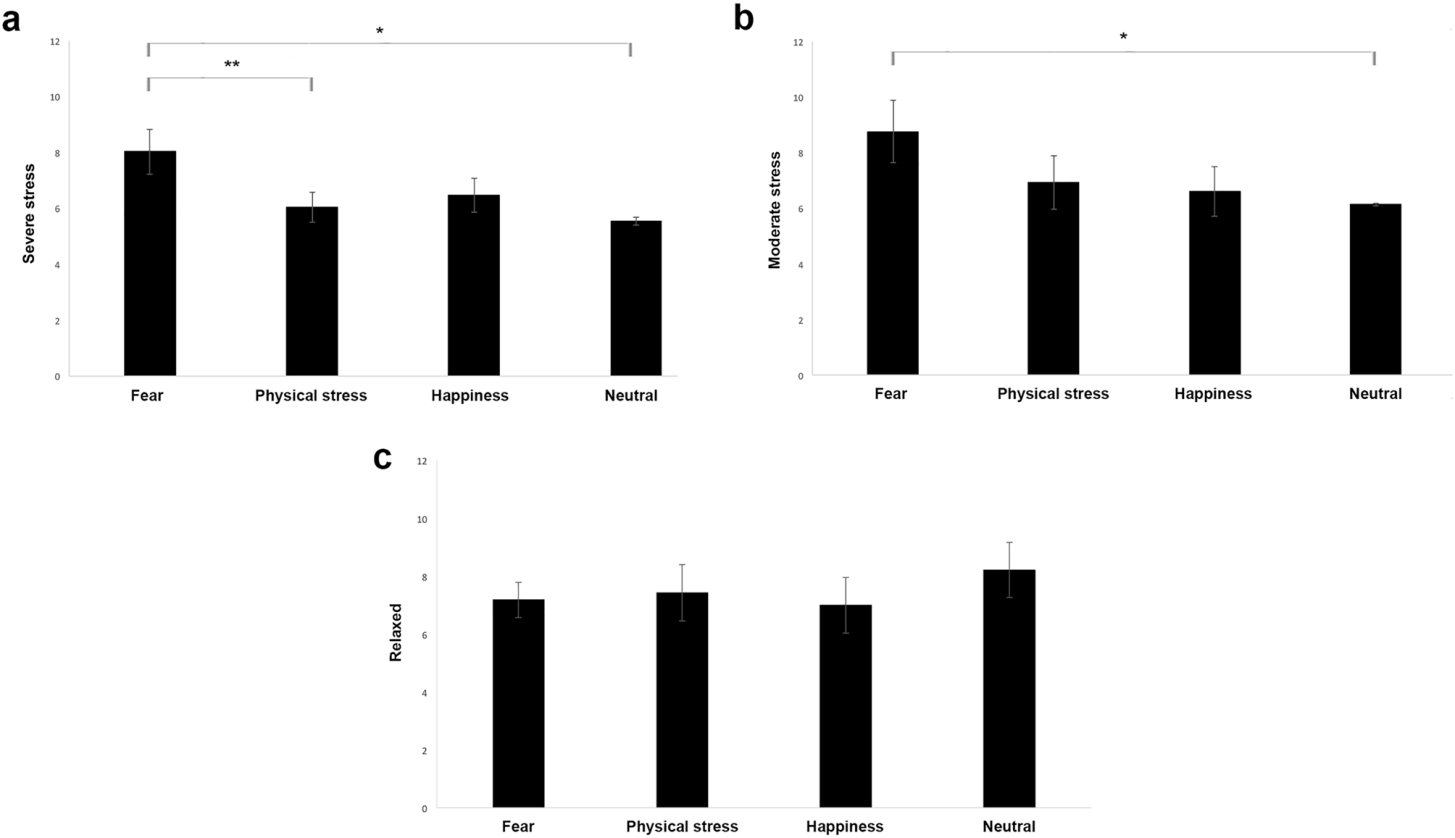
Behavioural responses to human emotional odours. Data of (**a**) severe and (**b**) moderate stress and (**c**) relaxed behavioural categories for the emotional odours (means ± s.e.m.). Cumulative mean, which were obtained from all the subjects during the emotional stimuli presentation (regardless the order of presentation), are shown. *p < 0.05; **p < 0.01.

As to behavioural data, the analysis of the severe stress behavioural category revealed significant differences between emotions (F(3,46) = 4.678, *P* < 0.01). The post hoc analyses showed that cats displayed more severe stress-related behaviour in response to “fear” odours than to “neutral” (P < 0.01; CI [0.83, 4.13]) and “physical stress” (P < 0.05; CI [0.05, 3.91]) (Fig. 2). No statistically significant differences of the severe stress levels for the other emotional odours were observed (“fear” vs. “happiness”:* P* = 0.140; CI [− 0.52, 3.61]; “physical stress” vs. “happiness”: *P* = 0.608; CI [− 2.14, 1.26]; “physical stress” vs. “neutral”: *P* = 0.384; CI [− 0.64, 1.64]; “happiness” vs. “neutral”: *P* = 0.171; CI [− 0.42, 2.28]). A significant emotion x order interaction was found (F(9,46) = 2.934, P < 0,01). It revealed statistically significant differences between “fear” and both “physical stress” (*P* < 0.05; CI [0.78, 10.24]) and “neutral” (*P* < 0.05); CI [1.02, 11.04]) when the stimuli were presented as first stimuli. A statistical significant difference was also found between cats’ severe-stress levels elicited by “fear” and “happiness” odours when presented as last stimuli; specifically, cats’ stress levels were higher in response to “fear” than “happiness” (P < 0.05; CI [0.85, 9.25]). No other statistically significant differences were identified (*P* > 0.05 for all other comparisons). Although the effect of emotion x order was found, no effect of the order regardless of the emotion presented was observed (F(3,46) = 0.758, *P* = 0.524). On the other hand, a statistical significant intervariability between subjects in their severe-stress levels was found (F(21,46) = 416.759,659, *P* < 0.001).

A significant effect of emotions on the moderate stress behavioural category was observed (F(3,51) = 2.933, *P* < 0.05). Post hoc analyses showed that the cats’ displayed more moderate stress-related behaviour in response to “fear” than “neutral” odours (P < 0.05; CI [0.37, 4.88]) (Fig. 2). No statistically significant differences of the moderate stress levels for the other emotional odours were observed (“fear” vs. “happiness”: *P* = 0.143; CI [− 0.75, 5.07]; “fear” vs. “physical stress”: *P* = 0.222; CI [− 1.14, 4.79]; “physical stress” vs. “happiness”: *P* = 0.804; CI [− 2.33, 2.99]; “physical stress” vs. “neutral”: *P* = 0.415; CI [− 1.16, 2.77]; “happiness” vs. “neutral”: *P* = 0.601; CI [− 1.33, 2.27]). Furthermore, a significant emotion x order interaction was revealed (F(9,51) = 2.689, P < 0.05): the analysis showed that the moderate stress levels were higher when cats were presented with “neutral” than “happiness” odours (P < 0.05; CI [0.32, 6.75]) when these stimuli were presented as second stimuli in the testing session; the cats’ moderate stress levels were higher in response to “happiness” than “physical stress” (P < 0.05; CI [1.05, 16.09]) when these emotional odours were presented as third stimuli; whereas moderate stress levels were higher for “fear” than “happiness” (P < 0.05; CI [1.99, 19.95]) and “neutral” (P < 0.05; CI [1.90, 19.19]) when these odours were presented as last stimuli. No other statistically significant differences were identified (*P* > 0.05 for all other comparisons). Although the effect of emotion x order was found, no effect of the order regardless of the emotion presented was observed (F(3,46) = 0.758, *P* = 0.524). On the other hand, the analysis revealed a statistical significant intervariability between subjects in the levels of moderate stress displayed (F(21,46) = 416.759,659, *P* < 0.001). Moreover, no significant differences between the severe and moderate stress for each emotion presented were observed (“fear”: Z = 137.50, P = 0.443; “physical stress”: Z = 74.50, P = 0.090; “happiness”: Z = 78.00, P = 0.603; “neutral”: Z = 66.00, P = 0.240; Wilcoxon signed-rank test).

A statistically significant intervariability between subjects was observed also with regard to the relaxed behavioural category (F(21,51) = 3.381 *P* < 0.001). No other significant differences with respect to relaxed behavioural category were observed (emotions: (F(3,51) = 0.383, *P* = 0.766); order (F(3,51) = 0.989, *P* = 0.405); emotion x order (F(9,51) = 1.281,* P* = 0.270).

Similarly, a statistically significant intervariability between subjects was observed for the LI (F(21,51) = 3.697, *P* < 0.001). No other significant differences with respect to LI were observed (emotions: (F(3,51) = 1.149, *P* = 0.338; order (F(3,51) = 0.185, *P* = 0.906; emotion x order (F(9,51) = 1.352, *P* = 0.235).

**Figure 3:**
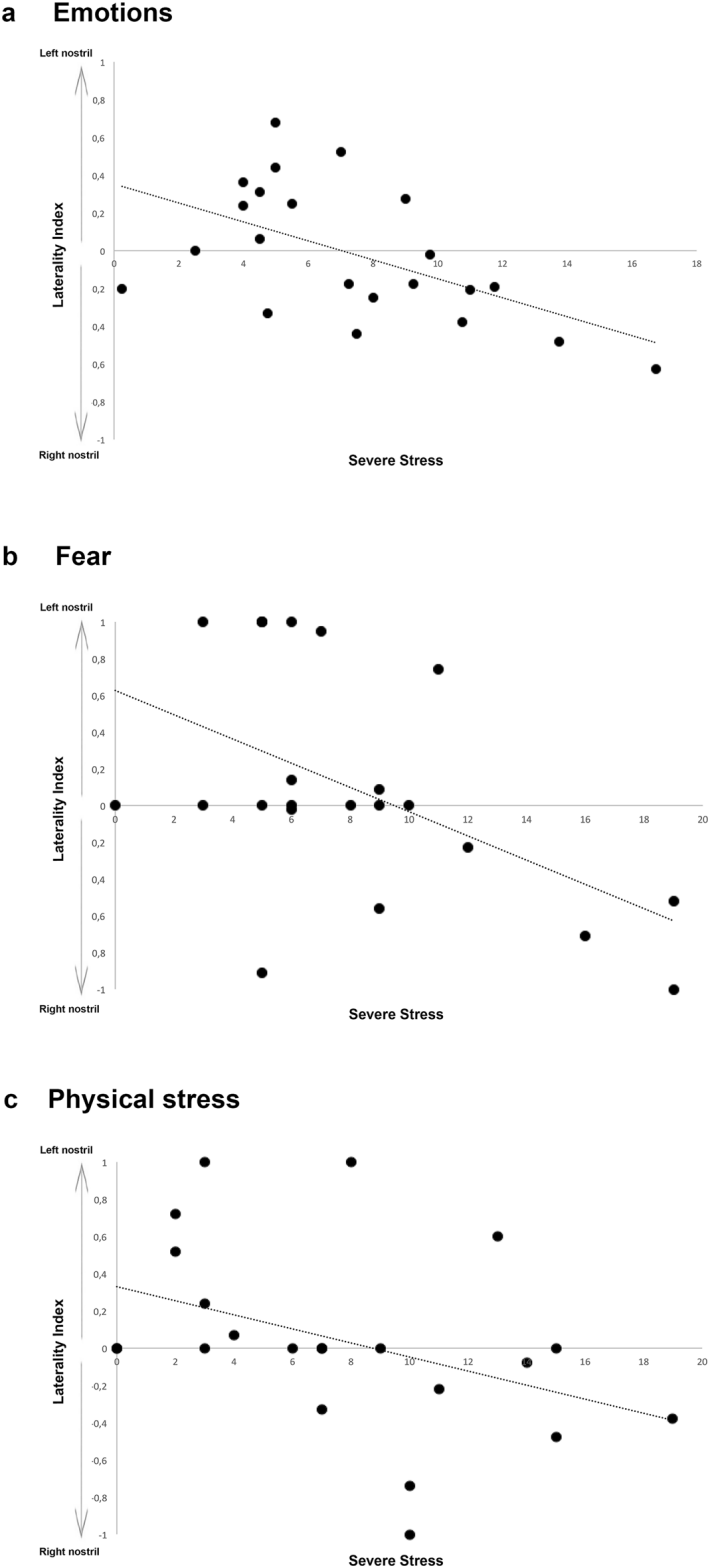
Relationship between Laterality Index (LI) and severe stress behavioural category. Correlation between the preferential use of the nostril (expressed by LI) and the individuals’ severe stress as cumulative data for (**a**) all the emotions presented (i.e., fear, physical stress, happiness, neutral), (**b**) fear and (**c**) physical stress.

We found no statistically significant bias in cats’ nostril preferential use when sniffing at the human emotional odours (One-sample Wilcoxon signed-ranks test: *P* > 0.05). However, a negative and statistically significant correlation was found between the LI and severe stress behavioural category (Pearson correlation: r22 =  − 0.534, *P* = 0.010); i.e. the higher the severe stress displayed by cats the more likely the right nostril was used to sniff odours (Fig. 3). Significant correlations were also found between the LI and the severe stress levels elicited by “fear” and “physical stress”. Specifically, a negative and statistically significant correlation was found between the LI and severe stress for “fear” and “physical stress” (Pearson correlation: “fear”: r22 =  − 0.464, *P* = 0.030; “physical stress”: r22 =  − 0.526, *P* = 0.012) indicating that the higher the severe stress displayed by cats in response to “fear” and “physical stress” odours, the more likely the right nostril was used to explore these emotional odours. In addition, a positive and statistically significant correlation was found between the LI and relaxed-related behaviours for “physical stress” (Spearman correlation: r22 =  − 0.505, *P* = 0.016) indicating that the higher relaxed-related behaviours displayed by cats in response to “physical stress”, the more likely the left nostril was used to sniff this emotional odour (Fig. 3). No other significant correlations between the LI and the relaxed, severe and moderate stress behavioural category for all the emotions analysed were found (*P* > 0.05).

**Figure 4:**
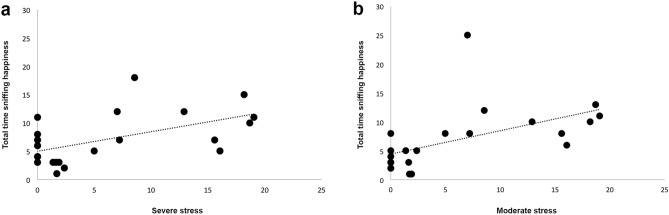
Relationship between the total time spent sniffing happiness odours and severe and moderate stress behavioural category. Correlations between the time spent sniffing happiness odours and the cats’ severe (**a**) and moderate (**b**) stress levels.

No statistically significant differences in the total time spent sniffing the swabs between the different emotions (N = 22, χ^2^ (3) = 2.355, *P* = 0.502; Friedman test). However, the analysis revealed that the total time spent sniffing “happiness” odour was positively correlated to moderate (Spearman correlation: r22 = 0.706, *P* = 0.000) and severe stress levels (Spearman correlation: r22 = 0.444, *P* = 0.038), indicating that the higher both moderate and severe stress displayed by cats in response to “happiness” odour, the longer the time spent sniffing this odour (Fig. 4). No other significant correlations were found between the total time spent sniffing the emotional odours and the behavioural categories analysed (*P* > 0.05).

Finally, a positive and statistically significant correlation was found between the cats’ age and the LI for “physical stress” (Pearson’s correlation: r22 = 0.451, *P* = 0.035), indicating that the higher the cats’ age, the more likely the left nostril was used. No other statistically significant correlations were found (*P* > 0.05 in all comparisons of age with LI and behavioural categories for all the emotions analysed).

## Discussion

Our results showed that cats are sensible to human emotional chemosignals conveyed by body odours, which induced different behavioural responses in the tested cats. In particular, “fear” odours elicited more stress-related behaviours than “neutral” (both moderate and severe stress) and “physical stress” (severe stress), suggesting that cats perceived the valence of the information conveyed by the “fear” olfactory signals. The negative correlation found between the severe stress and the laterality index further supports this hypothesis. It indicates indeed the prevalent use of the right nostril when the animals’ stress levels increased. Given that the mammals’ olfactory nerves ascend ipsilaterally to the brain^37^, the preferential use of the right nostril suggests the main involvement of the right hemisphere to process the olfactory stimulus. The right hemisphere controls the physiological and behavioural reactions to stressors, including the emotional ones, as widely reported in several domestic species (cats^35^; dogs^30,38^; horses^39,40^; cattle^41^; goats^42^). Therefore, although we failed to find statistically significant asymmetries in the nostril use to sniff human emotional odours, the prevalent use of the right nostril appears to be related to the increase of cats’ stress levels, particularly when the animals were presented with human “fear”. Interestingly, no significant differences in the cats’ stress levels were found between “happiness” and “fear”, suggesting that both the emotional odours produced an increase in cats’ arousal and their emotional activation. One possible explanation is that both the emotions were accompanied by an increase in the donors’ arousal level that could have been perceived by cats and enhanced their arousal state. Cats could have prioritized the processing of the chemosignals related to the donors’ arousal than those related to the valence of the emotion expressed. Being a predator but also a prey^36^, cats need to react fast to social and environmental stimuli that could threaten and lead them to adapt efficiently to changing conditions. It could be possible therefore that cats responded to human arousing chemosignals with increasing alertness due to the potential danger perceived. Alternatively, they could have faced difficulties in recognizing and classifying the emotional content of “happiness” odours in the absence of visual or auditory information, as previously shown for dogs^30,43^. The ambiguous valence attributed to the “happiness” odours is also suggested by the positive correlation found between the total time spent sniffing the swab and the animals’ stress levels, where a longer sniffing time corresponded to increasing stress levels (both moderate and severe). Previous studies reported that human olfactory signals are essential for eliciting emotional reactions in cats^23^ but they are insufficient for affecting cats’ emotional state when presented alone, i.e. without the human presence^44^. A multimodal representation of human emotions could be therefore needed for cats to clearly perceive the communicative content of human emotional signals. However, the possibility that the absence of significant differences between cats’ stress levels elicited by “fear” and “happiness” odours could be related to the limited sample size employed in our study cannot be entirely ruled out, suggesting the use of a larger population in future studies.

On the other hand, no differences in cats’ stress levels were found between “happiness” and “neutral” and between “neutral” and “physical stress”, suggesting that cats responded to human odours with a general increase in their arousal. Although all cats were socialized with humans and had a general positive attitude towards strangers, it could be possible that the odours of unfamiliar men could have elicited an initial and rapid alerting response. The lack of differences in the relax-related behaviours between the emotional odours appears to support this hypothesis. In addition, the possibility that the presence of an unfamiliar experimenter and the experimental setup might have affected the cats’ behaviour cannot be entirely ruled out, although the initial familiarization phase (i.e., before the beginning of the experiment) makes this hypothesis unlikely.

Overall, the different responses to “fear” odours, which significantly increased cats’ stress levels compared to “neutral” and “physical stress”, suggest that cats discriminate the content of human emotional odours. Contrary to previous studies, which report that cats are capable of distinguishing only their owners’ signals^5,23,27^, we found that cats generalize this ability to unfamiliar humans as reported by Humprey and colleagues^29^. It is of interest to note that a consistent intervariability between subjects in their emotional reactions to the human odours was observed, suggesting the influence of ontogenetic factors on the processing of human emotional signals in cats. This hypothesis is supported by previous studies showing that social experiences during life with humans, particularly in the early developmental periods, could impact cat sensitivity to human emotional signals^4^. Moreover, it has been found that human personality traits (i.e., agreeableness, conscientiousness, extroversion, neuroticism and openness), could mediate cats’ interspecific social interactions and the relationship between these species^21^. Another crucial role in the individual processing of emotional signals could also be played by the animals’ living conditions and welfare state given that a relationship between welfare and cognition has been widely reported in domestic animals (see for review^33^). For instance, previous studies indicate that horses showing poorer welfare state, which includes the presence of negative reactions to humans (i.e. aggressive behaviour toward humans), displayed a negative or “pessimistic-like” cognitive bias^45^, which impacts animals’ perception of the environment and social stimuli, including emotional signals.

Although we found no effect of the order of stimuli presentation on the cats’ behaviour, the interaction between the emotional odours and the order of the stimuli presentation appears to affect the cats’ stress level (both moderate and severe stress). In particular, the emotional stimuli having a marked difference in their arousal levels (i.e. “fear” vs. “neutral” and “physical stress”) appears to be less affected by the order of presentation than the stimuli having lower differences in their arousal levels, e.g. “happiness” and “fear”. The latter should be considered in future studies evaluating cats’ behavioural responses to emotional stimuli.

Contrary to dogs and horses^30–32,46^, we found no significant asymmetries in the cats’ nostril use while sniffing human emotional odours. Research over the years has shown that, although distinct, the main olfactory and the vomeronasal systems play an integrated role in detecting chemosensory cues, which mediate social behaviour^47^. Specifically, cats’ vomeronasal organ has more and diverse set of receptors than dogs’ (21 vs. 8) that have been shown to be involved in the analysis of social chemosignals^3^. Moreover, recent evidence shows that the vomeronasal system could detect some chemosignals perceived by the main olfactory system using the same receptor mechanisms^48^. Therefore, the apparent absence of asymmetries in cats’ nostril use during sniffing different human emotional odours could suggest a different perception and processing of such olfactory signals rather than the lack of functional laterality. An interesting hypothesis would be that after a first and general analysis of the main odour features by the main olfactory mucosa in terms of arousal, cats may engage in a secondary behavioural response that triggers vomeronasal organ cells for a finer and more detailed analysis of the odours in order to detect the individual emotional state. The presence of flehmen, licking and chewing behaviour directed toward the swabs, although anecdotally recorded in our study, point to the role of the vomeronasal organ in the perception of emotional signals in cats and merits future investigation.

The negative correlation between the laterality index and stress-related behaviours (both moderate and severe) observed in response to all the stimuli, which indicates the preferential use of the right nostril when cats stress level increased, supports the existence of lateralized emotional functions in cats’ brain. It is consistent with the right hemisphere specialization for arousing emotions and the processing of arousing stimuli that has been previously found in several species of vertebrates and invertebrates, including cats^33,35,49–51^. The presence of the same negative correlation for “fear” emotional odours together with the finding of a positive correlation between the laterality index and the relaxed-related behaviours for “physical stress” further confirms this hypothesis. The latter indicates the preferential use of the left nostril when the cats were relaxed. This result is in line with the left hemisphere dominant activity for low arousing emotional states^34^. Furthermore, a leftward bias for increasing cats’ age was observed for “physical stress” odours suggesting that adult pet cats mainly process this odour with the left hemisphere. Given the left hemisphere specialization for routine responses to familiar stimuli^33,34^, it could be possible that cats perceived the informant content of such odours as familiar due to their prior experiences and exposure to them.

Overall, our study revealed that cats are sensible to human emotional odours and regulate their behaviour accordingly. Moreover, our results provide first evidence of lateralized emotional functions of olfactory pathways in cats.

## Supplementary Information

Supplementary Information.

## Data Availability

The datasets generated and/or analysed during the current study are available from the corresponding author on reasonable request.
