# Supplementary material for: Relationship between asymmetric nostril use and human emotional odours in cats
Source: Sci Rep. 2023 Jul 6;13:10982. doi: 10.1038/s41598-023-38167-w (PMC10326042; doi:10.1038/s41598-023-38167-w)
Supplement: Supplementary file 1 — Supplementary Information. [file 41598_2023_38167_MOESM1_ESM.pdf]

Relationship between asymmetric nostril use and human emotional odours in cats

Serenella d'Ingeo[1*], Marcello Siniscalchi[1], Valeria Straziota[1], Gianluca Ventriglia[1], Raffaella Sasso[1] & Angelo Quaranta[1]

[1]Animal Physiology and Behaviour Unit, Department of Veterinary Medicine, University of Bari Aldo Moro, 70121 Bari, Italy.

*Correspondence: serenella.dingeo@uniba.it

VISUAL ANALOGUE SCALE

HAPPINESS

After watching the video, how do you feel?

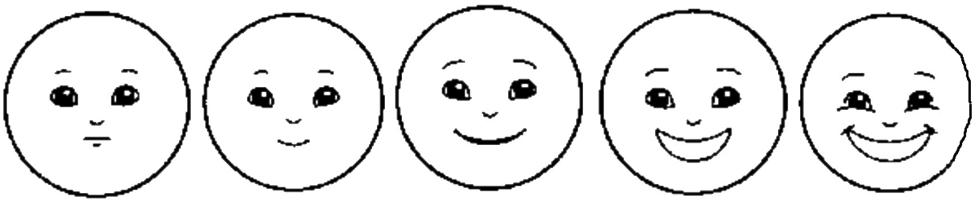

1 ☐ 2 ☐ 3 ☐ 4 ☐ 5 ☐

FEAR

After watching the video, how do you feel?

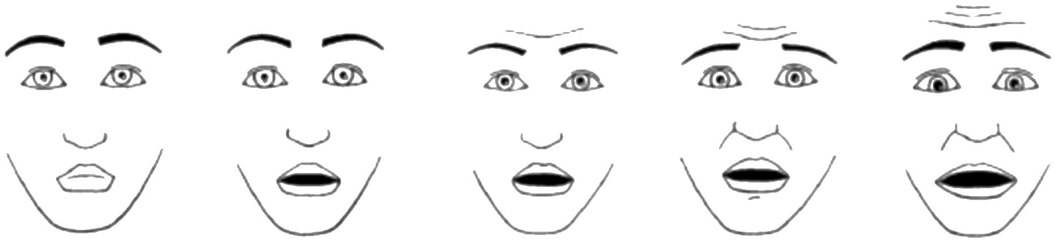

1 ☐ 2 ☐ 3 ☐ 4 ☐ 5 ☐

Figure S1. Visual analogue scale used to evaluate the donors' emotional response to "happiness" and "fear" eliciting movies [30].

**Behavioural category**
Severe stress
Move back
Fly away
Hiding
Crouched position
Arched back
Head back
Ears rotated backward/flattened
Eyes wide open
Tail held down and tense
Swishing tail
Moderate stress
Go away with tension in the body
Go to the owner with tension in the body
Standing tense
Resting tense
Sit tense
Ears sideward
Ears backwards
Dilatated pupils with normal open eyes
Half blink with dilatated pupils
Twitching tail tip
Relaxed
Go away with relaxed body
Go to the owner with relaxed body
Standing relaxed
Resting relaxed
Sit relaxed
Ears neutral (half-back)
Ears erected to front
Eyes soft
Half blink
Narrowed eyes
Extended or loosely wrapped/downward tail

Table S1. List of behaviours scored for behavioural analysis. Adapted from Bradshaw [36].

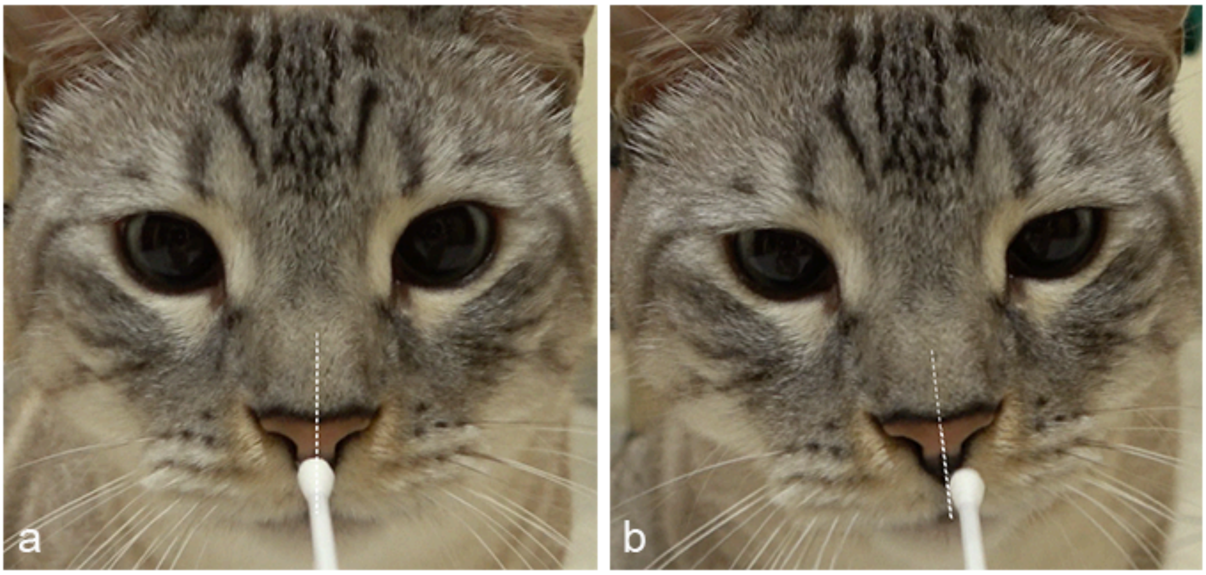

Figure S2. Cats' sniffing behaviour and nostril use: both nostrils (a) and left nostril preferential use (b) are shown (dotted line passing through the philtrum).
